# Clarifying the scope and capabilities of ROTS in differential expression analysis

**DOI:** 10.1093/bioinformatics/btag335

**Published:** 2026-05-22

**Authors:** Tomi Suomi, Jalmari Kettunen, Taneli Pusa, Laura L Elo

**Affiliations:** Turku Bioscience Centre, University of Turku and Åbo Akademi University, Turku, FI-20520, Finland; Turku Bioscience Centre, University of Turku and Åbo Akademi University, Turku, FI-20520, Finland; Turku Bioscience Centre, University of Turku and Åbo Akademi University, Turku, FI-20520, Finland; Turku Bioscience Centre, University of Turku and Åbo Akademi University, Turku, FI-20520, Finland; Institute of Biomedicine, University of Turku, Turku, FI-20520, Finland

## Abstract

**Summary:**

Recently, Anwar *et al.* introduced a method combining the ROTS reproducibility optimisation procedure with empirical Bayes variance estimation from limma. Here, we clarify several methodological aspects to support accurate interpretation of the results. We emphasise that ROTS is a general reproducibility optimisation framework rather than a single statistical test and demonstrate that benchmarking outcomes in the reported spike-in case studies are highly sensitive to analysis and evaluation choices. Furthermore, our reanalyses of the spike-in datasets do not support the reported conclusions, and we were unable to reproduce the results of the clinical Alzheimer’s disease case study. These findings highlight the importance of transparent benchmarking practices and careful interpretation of comparative results.

**Availability and Implementation:**

The ROTS package is available through Bioconductor. The reanalyses were performed using the original code, with the minimal additions described in the manuscript.

## 1 Introduction

Robust and reproducible differential expression results are essential in high-throughput omics studies to enable reliable biological interpretation and effective prioritisation of candidates for further validation experiments. The reproducibility-optimised test statistic (ROTS) was developed to address instability in feature rankings (e.g. genes or proteins) in high-dimensional differential expression analyses by optimising their reproducibility under resampling (Elo *et al.* 2008, Suomi *et al.* 2017). The ROTS framework, implemented in the R/Bioconductor package ROTS, has been widely used and has demonstrated strong performance in multiple independent benchmark studies (e.g. Fröhlich *et al.* 2022, Peng *et al.* 2024), in addition to our own benchmarking across diverse omics technologies (e.g. Seyednasrollah *et al.* 2016, Suomi *et al.* 2017, Suni *et al.* 2020).

In a recent article, Anwar *et al.* (2025) introduced LimROTS as a method for differential expression analysis that combines the ROTS reproducibility optimisation procedure with empirical Bayes variance estimation from the limma package and reported superior performance compared to ROTS (Anwar *et al.* 2025). However, to support accurate interpretation of both the reported method and its results, several aspects require clarification. We note that LimROTS is not part of the ROTS project and should not be considered a replacement of the existing ROTS framework.

## 2 ROTS is a general reproducibility optimisation framework

ROTS is a general framework for optimising a feature-ranking statistic by maximising reproducibility across resampled datasets. It is not tied to any specific variance estimator or statistic. While most published benchmarking of ROTS has focused on two-group comparisons, the framework supports a range of experimental designs, including multi-group analysis, survival analysis, and linear modelling that have been available in the Bioconductor ROTS package since version 1.5.1, 1.11.3, and 1.35.1, respectively. Incorporation of alternative estimators, such as the empirical Bayes variance estimate used in LimROTS, is compatible with the general ROTS optimisation principle and does not change the underlying reproducibility optimisation concept.

Consistent with LimROTS applying the same ROTS reproducibility optimisation procedure, Anwar *et al.* (2025) reported it to perform similarly as ROTS in typical two-group comparisons using spike-in proteomics datasets (case study 1).

## 3 Benchmarking results in spike-in case studies 2–4 are sensitive to analysis and evaluation choices

Case studies 2–4 in Anwar *et al.* (2025) were based on spike-in proteomics datasets involving batch effects. Key support for LimROTS was drawn from case studies 3 and 4, where synthetic batch effects were introduced by applying large shifts to a subset of proteins. While ROTS results for case studies 3 and 4 are not discussed directly in the manuscript, they are reported in the supplementary material and the package vignette, respectively (Anwar *et al.* Supplementary Table S3; LimROTS version 1.2.8 vignette, Bioconductor, 8 Dec 2025), with inconsistent treatment of batch effects: LimROTS is applied using batches as covariates in the models, whereas ROTS is applied without any batch adjustment.

**Figure 1:**
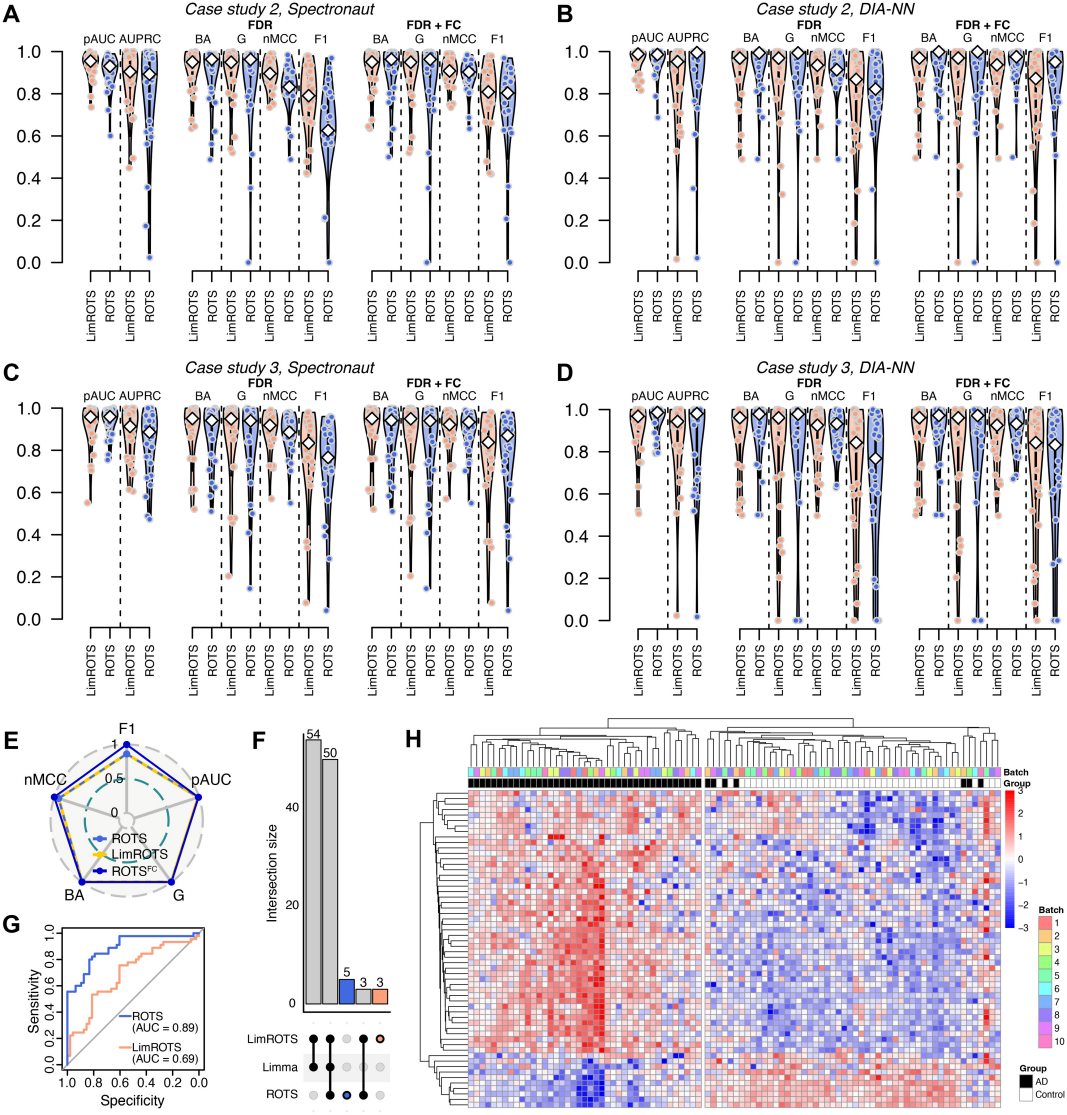
Reanalysis of the spike-in benchmark and clinical proteomics case studies. Performance of ROTS and LimROTS in case study 2 (different acquisition schemes) using (A) Spectronaut and (B) DIA-NN data, case study 3 (added synthetic batch effect) using (C) Spectronaut and (D) DIA-NN data, and (E) case study 4 (different software tools and added synthetic batch effect), with the diamond indicating the median across datasets in panels A-D. Performance is shown for the same evaluation metrics used by Anwar *et al*. (2025), including partial area under the ROC curve (pAUC) at 5% false positive rate, area under the precision-recall curve (AUPRC), arithmetic mean of specificity and recall (balanced accuracy, BA), geometric mean of specificity and recall (G-mean, G), normalised Matthews correlation coefficient (nMCC), and harmonic mean of precision and recall (F1 score). For metrics requiring a binary differential expression call, results are shown using both the original definition based on statistical significance alone (FDR < 0.05) as well as an alternative widely used definition requiring both statistical significance (FDR < 0.05) and effect size (minimum 1.5-fold change, FC). For case study 2, ROTS was used without batch adjustment, whereas for case studies 3 and 4, preprocessing-based batch correction was applied prior to differential expression analysis. LimROTS included batches in the models for all case studies 2–4. (F) Overlap of differentially expressed proteins in the clinical Alzheimer’s disease case study identified by ROTS, LimROTS, and limma at FDR < 0.05 and minimum 1.5-fold change. ROTS was applied after ComBat batch correction, while LimROTS and limma incorporated batch and other covariates in the models. (G) ROC curves and areas under the ROC curves (AUCs) from the predictive evaluation of generalised linear models with diagnostic status as the response and intensities of the uniquely identified significant proteins as predictors. (H) Heatmap of proteins identified as differentially expressed by ROTS in the clinical dataset, generated using the code provided by Anwar *et al*. (2025), illustrating separation between Alzheimer’s disease (AD) and control samples.

In case study 2, Anwar *et al.* (2025) combined data obtained using different acquisition schemes, yielding a confounding batch effect that was less extreme than the synthetic effects introduced in case studies 3 and 4. This case study was described as illustrating the importance of accounting for covariates, with LimROTS interpreted to outperform existing methods. However, the reported results do not support that LimROTS performs systematically better than ROTS across evaluation metrics and datasets, even with ROTS applied without batch adjustment (Fig. 1A and B).

Overall, the evaluation metrics had higher values for DIA-NN than for Spectronaut data (Fig. 1A and B). Notably, Anwar *et al.* (2025) used the global imputation method impSeq for the Spectronaut data and the left-censoring-based MinDet imputation for the DIA-NN data. In line with this, it has been shown that global imputation in the presence of batch-associated missingness can amplify random observations into systematic differences, whereas left-censoring-based approaches tend to be less prone to such artefacts (Hui *et al.* 2025).

In interpreting spike-in results, it is also important to note that each condition represents replicate runs of the same sample. Therefore, spike-in data mainly capture technical rather than biological variability and do not fully reflect the magnitude and structure of variation seen in real-world proteomics studies with biological replicates (Peng *et al.* 2024). To assess the robustness of the interpretations to the evaluation definition, we therefore additionally required a minimum 1.5-fold change together with the false discovery rate FDR < 0.05 to determine differentially expressed proteins. This is a commonly used definition in proteomics benchmarking that combines statistical significance and effect size (e.g. Lou *et al.* 2023, Peng *et al.* 2024) and was also used by Anwar *et al.* (2025) in the clinical case study. Under this definition, LimROTS and the established ROTS showed similar performance across evaluation metrics in both Spectronaut and DIA-NN data (Fig. 1A and B), with ROTS in the DIA-NN data producing the highest median values for all the four threshold-dependent evaluation metrics that require a binary differential expression call (Fig. 1B). These results indicate that the reported performance differences were not systematic but sensitive to data processing and evaluation choices.

## 4 Reanalysis of spike-in case studies 3 and 4 does not support the original conclusions

Proper handling of batch effects is widely recognised as crucial for reliable interpretation of omics data and has been repeatedly implicated in irreproducible findings and erroneous conclusions (Čuklina *et al.* 2021, Yu *et al.* 2024). A widely used approach is preprocessing-based batch correction, which is applicable with any two-group statistic (Johnson *et al.* 2007, Leek *et al.* 2012, Čuklina *et al.* 2021). In line with this, Anwar *et al.* (2025) did use preprocessing-based batch correction in the clinical case study. However, no batch adjustment was applied when reporting ROTS results for the spike-in case studies.

When we reanalysed the datasets of case studies 3 and 4 using preprocessing-based batch correction, we did not observe a performance advantage of LimROTS relative to ROTS. To ensure as direct a comparison as possible, we performed all analyses using the code provided by Anwar *et al.* (2025), and simply added a linear model-based batch correction from the limma package prior to differential expression analysis with ROTS. Additionally, ambiguities in FDR thresholding and pAUC calculation were corrected. Although ROTS can also incorporate batch directly as a covariate in the model, we used here preprocessing-based batch correction to provide a single strategy that remains applicable to any statistic.

In case study 3, Anwar *et al.* (2025) introduced an artificial batch effect to the case study 2 datasets affecting 500 proteins. Using the same datasets but adding preprocessing-based batch correction for ROTS, LimROTS did not show systematic performance advantage over ROTS across evaluation metrics and datasets (Fig. 1C and D).

The case study 4 dataset involved batch effects arising from different software tools and an additional simulated batch effect. Again, preprocessing-based batch correction for ROTS was sufficient to eliminate any apparent differences in performance (Fig. 1E). With the additional 1.5-fold change threshold, ROTS reached 1.0 across all five evaluation metrics used by Anwar *et al.* (2025) in this case study (Fig. 1E). These results suggest that the observed performance differences are substantially reduced when the data are handled in a consistent manner.

## 5 Reanalysis of clinical Alzheimer’s disease case study does not reproduce the reported results

Anwar *et al.* (2025) also reported performance differences between the approaches in a clinical dataset comparing post-mortem brain samples from Alzheimer’s disease patients (n = 48) and healthy controls (n = 45). In this dataset, Anwar *et al.* (2025) applied batch correction using ComBat (Johnson *et al.* 2007, Leek *et al.* 2012). However, the reported numbers of differentially expressed proteins for ROTS and limma differ between the main text, Supplementary File 2, and the accompanying Zenodo repository, making the originally reported relative performance of the methods difficult to interpret.

We therefore reanalysed the imputed protein intensity data provided by Anwar *et al.* (2025), adjusting for batch using ComBat in the same way as described in their workflow. ROTS identified 58 differentially expressed proteins at FDR < 0.05 and at least 1.5-fold change, the same thresholds used by Anwar *et al.* (2025). Limma identified 104 proteins, when using the same model as Anwar *et al.* (2025), with batch and other covariates included directly in the model. Comparison with LimROTS showed substantial overlap, with 50 proteins detected by all three approaches, five proteins unique to ROTS, three unique to LimROTS, and none unique to limma (Fig. 1F).

Further following the evaluation strategy of Anwar *et al.* (2025), we fit generalised linear models with diagnostic status as the response and protein intensities from the uniquely identified significant proteins as predictors. Under this evaluation, ROTS achieved a higher Cragg and Uhler pseudo R^2^ value (ROTS 57% vs. LimROTS 13%) and a higher area under the ROC curve (ROTS 0.89 vs. LimROTS 0.69, DeLong’s test *P* < 0.01; Fig. 1G). Similarly, using the code provided by Anwar *et al.* (2025), the heatmap based on the differentially expressed proteins identified by ROTS showed good separation between Alzheimer’s disease and control samples (Fig. 1H). Taken together, these reanalyses do not provide consistent evidence for a systematic performance advantage of LimROTS over ROTS.

## 6 Conclusions

Benchmarking in computational biology is known to be sensitive to design choices, from dataset selection and preprocessing to method settings and evaluation criteria, and self-authored benchmarks often report more optimistic performance than independent evaluations (Norel *et al.* 2011, Buchka *et al.* 2021). Our reanalysis suggests that the reported performance differences between the two methods may be sensitive to analysis and evaluation choices, including the handling of confounding batch effects across the compared analyses. When standard preprocessing was applied in line with common practice, we did not observe a systematic performance advantage of LimROTS over ROTS across the spike-in and clinical datasets. In case study 2, relative performance difference was not systematic across evaluation criteria and datasets even with ROTS applied without batch adjustment (Fig. 1A and B), and in case studies 3 and 4, applying a consistent batch correction strategy removed any apparent differences (Fig. 1C and E). In the clinical dataset, the reported advantage of LimROTS was not reproduced when following the stated workflow using the provided data, while downstream evaluations favoured ROTS instead (Fig. 1G and H).

Finally, we would like to emphasise the value of clearly situating incremental extensions within existing frameworks, with transparent credit to the original work and a clear explanation of how new components relate to established tools. ROTS is a general and flexible framework that accommodates a wide range of data types and experimental settings. LimROTS builds on this framework and its implementation but is distributed as a separate package under a closely related name, which may make it harder for users to distinguish between the original method and its derivative. We hope that these clarifications help interpret the comparative analyses in Anwar *et al.* (2025) and select appropriate tools for reproducible differential expression analysis. Of note, the functionalities described as future developments for LimROTS, including C++-based optimisation and parallelisation, are already available in the Bioconductor ROTS package.
